# Topological data analysis gives two folding paths in HP35(nle-nle), double mutant of villin headpiece subdomain

**DOI:** 10.1038/s41598-022-06682-x

**Published:** 2022-02-17

**Authors:** Takashi Ichinomiya

**Affiliations:** 1 Department of Systems Biology, Gifu University School of Medicine, Yanagido 1-1, Gifu, 501-1194 Japan; 2 The United Graduate School of Drug Discovery and Medical Information Sciences of Gifu University, Yanagido 1-1, Gifu, 501-1194 Japan

**Keywords:** Biological physics, Applied mathematics, Computational biophysics, Computational science

## Abstract

The folding dynamics of proteins is a primary area of interest in protein science. We carried out topological data analysis (TDA) of the folding process of HP35(nle-nle), a double-mutant of the villin headpiece subdomain. Using persistent homology and non-negative matrix factorization, we reduced the dimension of protein structure and investigated the flow in the reduced space. We found this protein has two folding paths, distinguished by the pairings of inter-helix residues. Our analysis showed the excellent performance of TDA in capturing the formation of tertiary structure.

## Introduction

The folding mechanism of protein is a primary area of interest in protein science. Understanding the process of protein folding is essential for recognizing the role of proteins in living cells and developing new therapies for protein-misfolding diseases such as Creutzfeldt-Jakob disease or Alzheimer’s disease^1^. The development of computer technology has enabled large-scale molecular-dynamics(MD) simulations for this purpose. However, it remains challenging to obtain essential information on the folding process from data obtained using MD.

MD simulation provides extensive data, which must be condensed to obtain essential information. Previous studies have attempted to identify the essential bonding that characterizes protein folding structure by trial and error. Data-scientific methods, such as principal component analysis (PCA) or k-means clustering have also been used to study the folding structures. PCA has provided successful results in analysis of folding dynamics^2^. However, the nonlinearity and nonlocality of the folding process often make it difficult to apply such methods. In protein folding, a small change in the local bending angle causes a large change in the position of all residues; linear analysis, such as PCA or k-means, sometimes provide insufficient information. To overcome this difficulty, complex procedures are often needed. For example, Jain and Stock used a combination of dihedral PCA, k-means clustering, Markov state modeling, and hierarchical clustering to analyze the folding of protein HP35^3^. Another approach is the application of non-linear analysis, such as kernel PCA, isomap, or t-distributed stochastic neighbor embedding. For example, Das et al. reported that isomap gives extremely smaller residual variance than PCA^4^. However, these nonlinear analysis methods require large computational costs and are not applicable when the number of samples exceeds $O(10^4)$.

Recently, topological data analysis(TDA) has attracted considerable attention in a variety of fields^5^ . In TDA, the structure of the data is characterized using topological features, such as the number of loops or connected components. TDA has several advantages compared to other data analysis methods for protein folding; first, it can capture the nonlocal characteristics in the data. In the standard analysis of protein folding, Cartesian coordinates or the dihedral angle of residues are used to characterize the structure, but these variables are defined by one, two, or three residues. TDA captures structures such as loops or cavities, which are defined as the set of many atoms or residues, and is suitable for capturing nonlocal structures. Second, TDA has an advantage in capturing “unfolded states”. Usually, the unfolded state of a protein has a large degree of freedom in motion because the chain of residues can bend almost freely. This large degree of freedom often makes standard analysis methods such as PCA or k-means clustering difficult because they are strongly affected by the large fluctuation in this state. However, in the unfolded state, there are no or few stable loops, and the unfolded state can be labeled as a state without a loop in the TDA. Third, TDA provides intuitive insights because the “loop” or “cavity” can be intuitively recognized; thus, TDA gives comprehensible information on the structure.

In the last decade, several studies have attempted to apply TDA for the analysis of biomolecules, such as RNA or proteins. For example, Yao et al. analyzed the folding path of RNA using Mapper, a popular TDA method^6^. Xia and Wei proposed “topological fingerprints” to classify protein structures^7,8^. They also attempted to apply the combination of deep learning and TDA to classify the protein structure^9^. In our previous study, we proposed a feature construction method based on persistent homology (PH), the most widely used TDA method^10^. We applied this method to the MD simulation of chignolin and identified stable, meta-stable, and transition states.

In this study, we applied our method to the dynamics of HP35(nle-nle), a double mutant of the villin headpiece. HP35 is a small protein consisting of 35 residues; it has three $\alpha$-helices and is one of the first proteins whose folding processes are reproduced by MD simulations. However, the folding process of this protein is not completely understood. After the success of MD simulation by Beauchamp et al.^11^, several groups have investigated the folding process of proteins in this group^12–14^. Although several experimental studies have suggested the existence of multiple folding paths, the theoretical results are controversial. After Piana et al. showed that $\alpha$-helix 3 was generally formed first in this protein^12^, Harada et al. presented that there are two folding paths, distinguished by the order of the formation of $\alpha$-helices^13^. Wang et al. claimed to have identified a third path^14^. In these preceding studies, the paths are classified by the order of formation of $\alpha$-helices. However, it is not clear whether this order results in a different tertiary structure formation process. In this study, we analyzed the MD data f HP35(nle-nle) obtained by Beauchmap et al. using TDA. The results showed that there are two folding paths, characterized by the pairings between V9 and K32, F10 and L28, F17 and L20, L20 and Q25, and F17 and Q25. These parings are inter-helix coupling and suggest the existence of multiple paths that have different tertiary structures.

## Methods

### Data preprocessing

**Figure 1:**
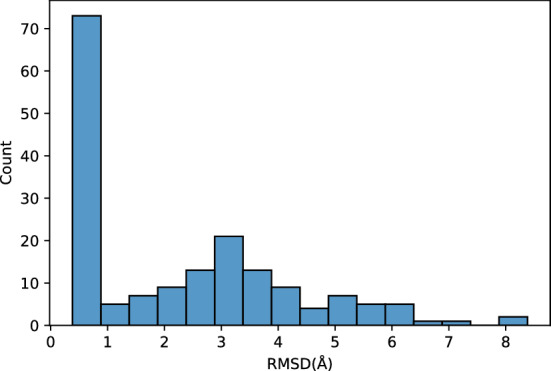
Distribution of minimum root mean square distance between trajectories and native state.

The dataset provided by Beauchmap and Pande downloaded from Stanford University (https://exhibits.stanford.edu/data/catalog/bd829sf1034) was used. This dataset was created by the massive distributed computing system Folding@home^15^ and has 175 trajectories. Each trajectory was named as “RUN”+integer, such as RUN897 or RUN45767. Every trajectory has snapshots of atoms every 250 ps. The length of trajectories are not the same, and 1,352,345 snapshots were obtained in total. Before TDA, these trajectories were investigated and it was found that some trajectories failed to fold correctly. For example, when we calculate the root mean square distance(RMSD) between C$_\alpha$ atoms in the native state and snapshots in trajectory RUN3693, the minimum of RMSD was 8.044 Å. We analyzed the dataset including non-folding trajectories by the procedure described in the following subsection, but failed to determine the appropriate rank for dimension reduction. Unfolded data will give important insights on the protein dynamics; however, we concentrated on the folded trajectories in this study to reveal the process of correct folding. To remove unfolded trajectories, we calculate the RMSD between the native state and every snapshot for all trajectories and calculated the minimum of RMSD for all trajectories. The distribution of the minimal RMSD is shown in Fig. 1. This figure shows that many trajectories were not correctly folded. Therefore, the trajectories whose minimum RMSD from native state is smaller than 1.0 Åwere selected. Moreover, all atoms had the same Cartesian coordinates in several snapshots; for example, the position of all atoms in RUN48080 is (22.500, 22.500, 22.500) for $t\ge 1700750$ ps. These unreliable data appeared at the end of several trajectories, which may have been caused by errors in merging the results of distributed computation. These snapshots were removed and 74 trajectories with 575,968 snapshots were obtained. These data were used in the subsequent TDA.

### Persistent homology analysis

To analyze the structure of protein, a PH analysis of the point cloud of residues was performed. Although PH can be formulated in pure mathematical framework^16^, here, PH is explained based on $\alpha$-filtration with degree 1, used in the analysis.

**Figure 2:**
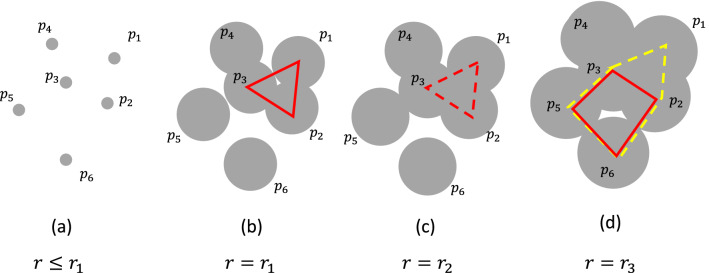
Schematic explanation of PH. (**a**) When the radius of balls *r* was small, all balls were disconnected. (**b**) When *r* increased, a cycle that surrounds the empty space is created, as indicated by the red triangle. (**c**) A further increase in *r* destroyed the cycle. (**d**) Several polygons may surround the empty space, as shown by the yellow and the red polygons. The volume optimal cycle was defined as the minimal cycle that surrounded the empty space. In this case, the red and yellow polygons consisted of two and three triangles, respectively, and the red polygon is the optimal one.

First, $p_1=(x_1, y_1, z_1),p_2= (x_2, y_2, z_2), \ldots , p_N=(x_{R}, y_{R}, z_{R})$, the positions of C$_\alpha$ atoms in protein were taken as inputs, where *R* represents the number of residues. Next, we put balls with radius *r* at $p_1, p_2,\ldots , p_N$. If *r* was small, all atoms were disconnected, as shown in Fig. 2a. By increasing *r*, the residues began to merge and a “cycle” surrounding the empty space formed at $r=r_1$, as indicated by the red triangle in Fig. 2b. However, the cycle shrank by the further increase of *r* and disappeared at $r=r_2$ as indicated by the red dashed triangle in Fig. 2c. PH theory guarantees that every cycle that emerges and disappears when we increase *r* from 0 to $\infty$ can be characterized by its birth *b* and death *d*. In this example, the atomic configuration has a cycle with $(b,d)=(r_1, r_2)$. In PH analysis, we used the set of births and deaths of cycles to characterize the structure of the data set. In our approach, the “volume optimal cycle” *C* was also used, depicted by the red polygon in Fig. 2b,d. The volume optimal cycle was defined as the “minimal” cycle that surrounds the empty space when the cycle emerges^17^. For example, in Fig. 2d, both red and yellow polygons surround the empty space. However, the red polygon is the sum of two triangle, while the yellow polygon is sum of three. Therefore red polygon is “smaller” than yellow one. In this case, red polygon represents the volume optimal cycle. We note that every cycle can be uniquely determined by the list of pair of atoms; for example, the volume optimal cycles in Fig. 2b,d are represented as $\{(p_1, p_2), (p_2, p_3), (p_1, p_3)\}$ and $\{(p_2, p_3), (p_3, p_5), (p_5, p_6), (p_2, p_6)\}$, respectively. In this study, we list up all cycles when we change $r=0$ to $\infty$, and use the set of triplet (*b*, *d*, *C*) as the features of the dataset.

In this study, several assumptions regarding the PH of proteins were made. First, we use PH with degree 1, which provides information on the “loop”. PH with different degrees provides other topological information, such as the number of connected components or cavities. However, loop information is especially useful for characterizing the structure of proteins^7,10^. For example, the formation of $\alpha$-helices or $\beta$-sheets can be characterized by the formation of a loop, while information on connected components or cavities does not provide insight into these structures. Therefore, information of PH with degree 1 was used. Second, each loop was characterized by birth and death in PH, and “lifetime,” the difference between death and birth, was also considered as an important variable in PH, because it is related to the robustness against noise^18^. The cycle that emerged due to the small perturbation of atoms may have large birth or death values, but always have a small lifetime. Therefore, the lifetime was used for the vectorization explained in the next subsection. Third, although most of the applications of PH mainly use births, deaths, and lifetimes to characterize the structure, the volume optimal cycle was also used, because births, deaths, and lifetimes were insufficient to characterize the structure of HP35(nle-nle). One of the limitations of standard PH is that it cannot distinguish between similar structures. For example, the native state of HP35(nle-nle) has three $\alpha$-helices. Unfortunately, the cycles that emerged from these helices had similar births and deaths. Therefore, births and deaths provided insufficient information to distinguish the process of $\alpha$-helix formation. The volume optimal cycle provided the shape and location of loops, and helped to distinguish the order of $\alpha$-helix formation. The calculation of births, deaths, and volume optimal cycles were carried out using HomCloud 2.8.1^19^.

### Vectorization of volume optimal cycles by “bag of simplices”

**Figure 3:**
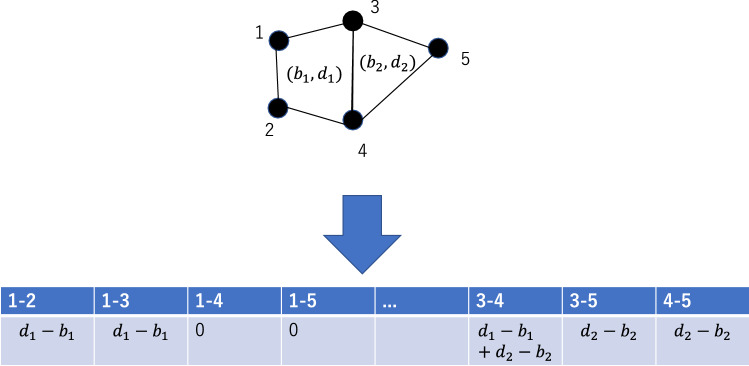
Definition of “bag of simplices”.

For machine learning, information obtained by PH must be vectorized. Here, “bag of simplices”, inspired by “bag of words” that is frequently used in natural language processing, was used^10^. In this approach, the set of volume optimal cycles was regarded as a “text” that describes the structure of a protein. Suppose that the protein structure had volume optimal cycles $C_1, C_2,\cdots C_{N_c}$. As we have noticed, every volume optimal cycle could be represented as a set of pairs of atoms, $C_k = \{(i_{k_1},j_{k_1}),(i_{k_2}, j_{k_2}), \cdots , (i_{k_l}, j_{k_l})\}$. $s_{i,j}$ was defined as the “weight” of the edge between *i* and *j*, as

1 $$\begin{aligned} s_{ij} = \sum _{C_k \ni (i,j)} (d_k - b_k), \end{aligned}$$

where $d_k$ and $b_k$ are the death and the birth of cycle $C_k$, respectively. Using this method, the volume optimal cycles were converted into $\frac{R(R-1)}{2}$-dimensional vectors. This procedure is shown in Fig. 3.

### Dimension reduction by non-negative matrix factorization

In the case of HP35(nle-nle), the feature vector obtained as a bag of simplices had $35\times 34/2=595$ dimension. The dimension was so large that dimension reduction was needed to obtain comprehensible information. For this purpose, non-negative matrix factorization (NMF) was used^20^. Suppose that there were *M* samples whose *N*-dimensional feature vector was given by a non-negative vector $\user2{v}_i=(v_{i1}, v_{i2},\ldots ,v_{iN})^T$, where *i* denotes the index of the sample. In NMF, the non-negative $N\times L$ matrix $\user2{W}$ and $L\times M$ matrix $\user2{H}$ that minimizes $||\user2{V}-\user2{WH}||$, where $\user2{V}= (\user2{v}_1, \user2{v}_2, \ldots , \user2{v}_M)$, and $||\cdots ||$ represents the Frobenius norm, were calculated. Using NMF, the approximation $\user2{v}_i \sim \sum _{j=1}^L \user2{w}_j h_{ji}$ was obtained, where $\user2{w}_j$ represents *j*-th column of matrix $\user2{W}$.

Compared with other dimension-reduction techniques, such as PCA, manifold embedding, or deep learning, NMF has several advantages. First, it is easy to interpret the results of the dimension reduction. In PCA, the feature vectors were approximated by a linear combination of several vectors. This was similar to NMF, but negative components and coefficients in PCA often made it difficult to interpret the results. When attempting to approximate the feature vector *v* using PCA, some components may become negative. If *v* must be a non-negative vector by definition, careful treatment is required to interpret the result. All coefficients and bases were non-negative in NMF, and a negative component never appeared in the approximated feature vectors. Second, as shown by Lee and Seung , NMF often captures “local” structure, while PCA captures global structure^20^. This property, which has not been proven rigorously, makes it easy to interpret the results. Third, the computational cost of NMF is low. NMF can be carried out at a lower computational cost than nonlinear analysis, such as manifold learning or deep learning.

Of course, NMF also has several disadvantages. First, NMF decomposition is not unique. If both *A* and $A^{-1}$ are $L\times L$ non-negative matrices, then $W^\prime = WA$ and $H^{\prime } = A^{-1} H$ are also non-negative, and another NMF is obtained. In particular, the scale of *H* and *W* changed if *A* was diagonal matrix, $A=\text{ diag }(\lambda _1, \lambda _2,\ldots , \lambda _L)$. NMF analysis was carried out several times and the consistency was checked in this study, and the result was rescaled to satisfy $|\user2{w_i}| = 1$ for $i=1,2,\ldots , L$. Therefore the unit of NMF scores presented in next section is Å. Another problem in NMF is that there is no standard rule for determining the appropriate rank *L*. In this study, we performed two methods to estimate the appropriate rank. The first one, proposed by Hutchins et al.^21^, uses the residual sum of squares (RSS) to determine the appropriate rank. In this method, we investigated the rank dependence of RSS between $\user2{V}$ and $\user2{WH}$, and chose *L* at the inflection point. Another one, proposed by Brunet et al.^22^, uses the cophenetic correlation coefficients for rank determination. In this method, we carry out hierarchical clustering after NMF several times. As the results of NMF depend on the random initial condition, the results of clustering are different each time. In this approach, we calculated the cophenetic correlation coefficients, which indicate the consensus among clustering, and choose the rank that gives high cophenetic correlation coefficient. Unfortunately, these methods require high computational costs. For cost reduction, we randomly chose 10,000 samples from the dataset, and calculated the RSS and cophenetic correlation for $L=3, 4, \ldots , 10$.

In this paper, the calculation of RSS and cophenetic coefficients was carried out using R 4.1.2^23^ and package NMF 0.23.0^24^. After determining the rank, the remaining analysis was performed using scikit-learn 0.24.1^25^.

## Results

### Density of states in reduced space

**Figure 4:**
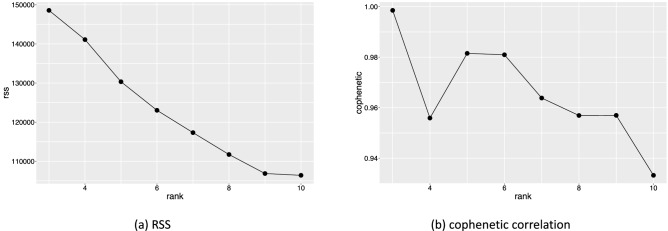
Rank dependence of (**a**) residual sum of squares(RSS) and (**b**) cophenetic coefficients.

First, we showed the plot of RSS and cophenetic coefficients to determine the appropriate rank. Figure 4 shows the rank dependence of RSS and cophenetic coefficients. Figure 4a shows that RSS decreases almost linearly with rank, and we cannot determine the appropriate rank. Cophenetic correlation in Fig. 4b shows a peak at rank $L=5$ or 6. We carried out NMF decomposition for $L=4$, 5, and 6, and we found no quantitative changes. In the following, we show the result for $L=5$.

**Figure 5:**
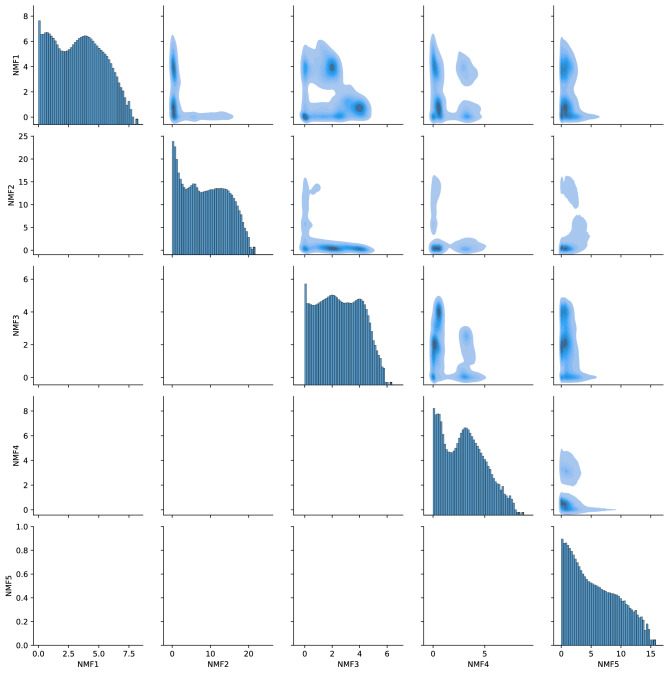
Density of states in reduced space by non-negative matrix factorization (NMF). The unit of each axis was Å. Figures in diagonal part shows the logarithmic histogram of each variables.

Figure 5 shows the density of states in reduced space obtained by NMF for $L=5$. First, all histograms in the diagonal of Fig. 5 had large strong peaks at 0. This strong peak was also found in the case of chignolin, and was related to the unfolded state^10^. In the unfolded state, the number of cycles was small, and these cycles had a short lifetime. Therefore, almost all components of the “bag of simplices” vector became zero in the unfolded state, which resulted in a strong peak at 0 in the histogram. Second, the distribution of the scores of NMF1, NMF3, and NMF4 had two other peaks. The density of the states represented in the upper right triangle of Fig. 5 shows that there are several high density peaks in the space constructed from these three components, while the distribution of NMF2 and 5 did not show a clear structure. This observation was not modified when we changed the embedding dimension *L* to 4 or 6; and we only considered the reduced space spanned by NMF1, NMF3 and NMF4. Here, we note that the density obtained by this analysis was not directly related to the free energy. In the dataset, the simulation began from the unfolded state and stopped before reaching thermal equilibrium. Therefore, the densities shown in Fig. 5 do not reflect the free energy, though the high-density state may be related to the meta-stable structure.

**Figure 6:**
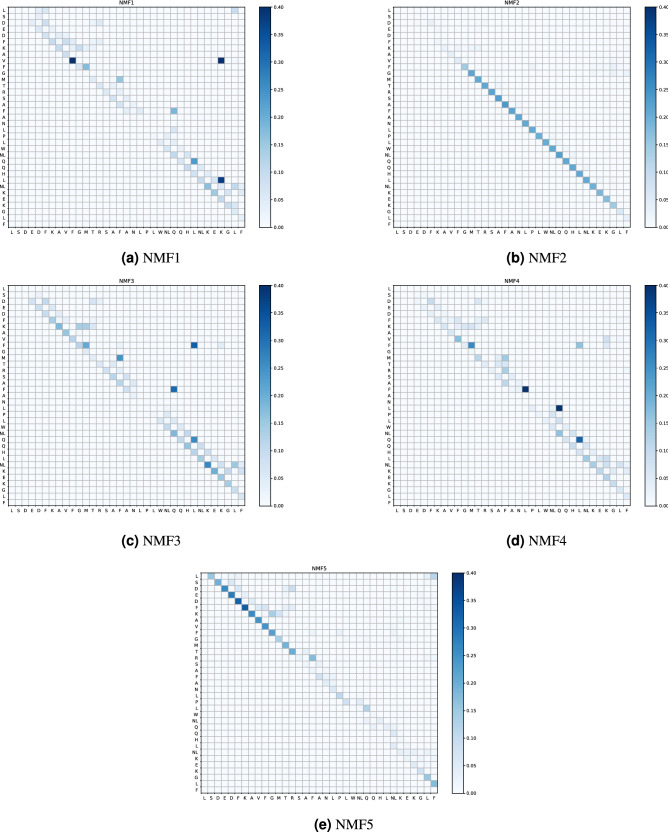
Basis of non-negative matrix factorization(NMF). Darker cells indicate that the basis has a large component in this cell.

To identify the meaning of each component, the basis that corresponded to each component is shown in Fig. 6. In this figure, the value of each component is visualized by color. For example, in Fig. 6a, the block at V9 and K32 is dark; therefore, the basis of NMF1 has a large value at V9–K32. Therefore, if a sample had a large NMF1 score, there were large loops that included the link between V9–K32. In the case of PCA, this conclusion should be reached carefully, because there may be a neglected basis that has a large negative value at V9–K32. In the case of NMF, every basis provides only a positive contribution, and we do not need to consider such a case.

**Figure 7:**
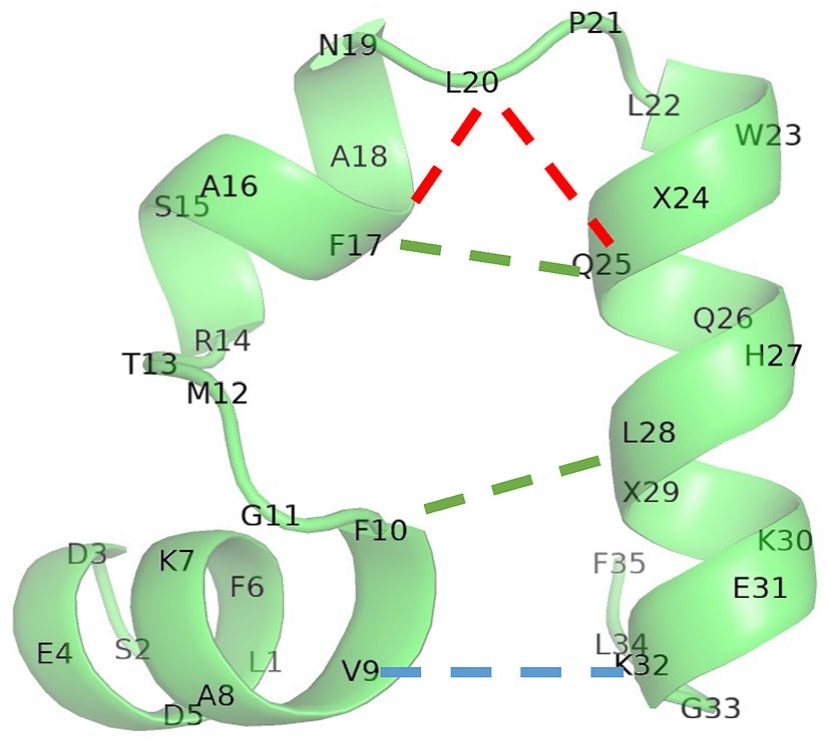
Native state of HP35(nle-nle). Blue, green, and red dashed lines represent the pair whose component was larger than 0.3 in the 1st(blue), 3rd (green), and 4th (red) components obtained by non-negative matrix factorization, respectively. Here, we omitted the pairs inside the $\alpha$-helices. X represents norleucine.

From Fig. 6, we first found that the vectors except NMF2 showed similar pairing between 3-residue-apart residues, such as D3–F6 or Q25–L28. These pairs implied the formation of an $\alpha$-helix. In the native structure, as shown in Fig. 7, these residues were close to each other along $\alpha$-helices. However, NMF2 did not show these pairs. Therefore, if an $\alpha$-helix is formed, the scores of NMF1, 3, 4 and 5 increased, while those of NMF2 increased when the loop without the $\alpha$-helix is formed. Second, the components of NMF1, 3 and 4 had pairs that characterized each state. For NMF1, the component at V9–K32 exceeded 0.3. Therefore, if there was a loop that involved these two residues, NMF1 score increased. Similarly, for NMF3, F10–L28 and F17–Q25 exceeded 0.3, and the NMF3 score increased when there were loops that included these edges. NMF4 score increased when there were loops with F17–L20 or L20–Q25. This suggestion is also supported by the correlation between NMF scores and weight of pairs in the bag of simplices, as shown in Table 1. The correlation between NMF1 score and weight of V9–K32 was 0.926, which suggests a strong correlation between these two variables. Similarly, NMF3, F10–L28, F17–Q25, NMF4, F17–L20, and L20–Q25 showed strong correlations, which supports our suggestion. To recognize the structure associated with these characteristic pairs, we showed these pairs in the native state in Fig. 7. Overall, these pairs appeared to represent the formation of a tertiary structure. As shown in Fig. 7, V9–K32 and F10–L28 pairs represented the contact between two $\alpha$-helices at the terminal subdomains. Pairs F17–L20, F17–Q25, and L20–Q25 represented the bending between second and C-terminus helix. NMF5 has no components that characterize its structure. Therefore, the large NMF5 implied that there is no clear tertiary structure, although $\alpha$-helices may exist.

**Table 1:** Correlation between NMF1, NMF3 and NMF4 scores and weights on edges.

	V9–K32	F10–L28	F17–Q25	F17–L20	L20–Q25
NMF1	0.926	− 0.420	0.350	− 0.067	0.010
NMF3	− 0.366	0.668	0.570	− 0.394	− 0.348
NMF4	− 0.030	0.160	− 0.633	0.953	0.861

### Dynamics in reduced space

**Figure 8:**
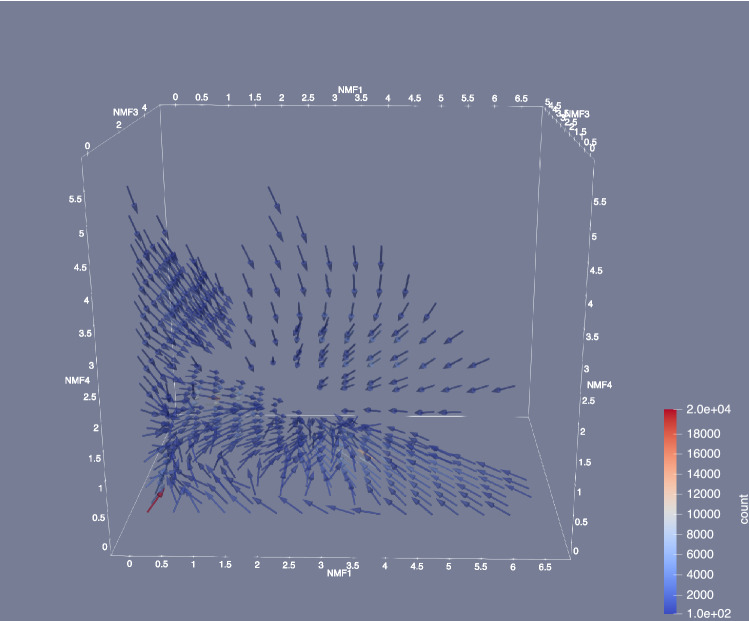
Average direction of flow of the systems in reduced space. The colors or arrows represent the number of samples in each cell. Only the flows of cells that include more than 100 samples are shown. The lengths of the arrows are not scaled, because the velocity at (NMF1, NMF3, NMF4) $\sim (0,0,0)$ was too small, thus, invisible.

We then analyzed the dynamics in the reduced space. We divided the 3D space spanned by NMF1, 3, and 4 into $20\times 20\times 20$ cells, and calculated the mean velocity of the systems in each cell. Although the average velocity gives limited information as we discuss later, it gives intuitive information on the dynamics. The flow direction is depicted in Fig. 8. In this figure, only the flows of cells that include more than 100 samples are shown. This figure shows that there is an attractive fixed point at (NMF1, NMF3, NMF4) $\sim (3.0, 3.0, 1.0)$. Interestingly, the path to this fixed point was not linear. For example, when we investigated the direction of the flow when NMF3 and 4 were small, the flow headed to the fixed point for NMF1 $\gtrsim 4$, whereas it was directed to the position (0,0,0) when NMF1 $< 3.0$.

**Figure 9:**
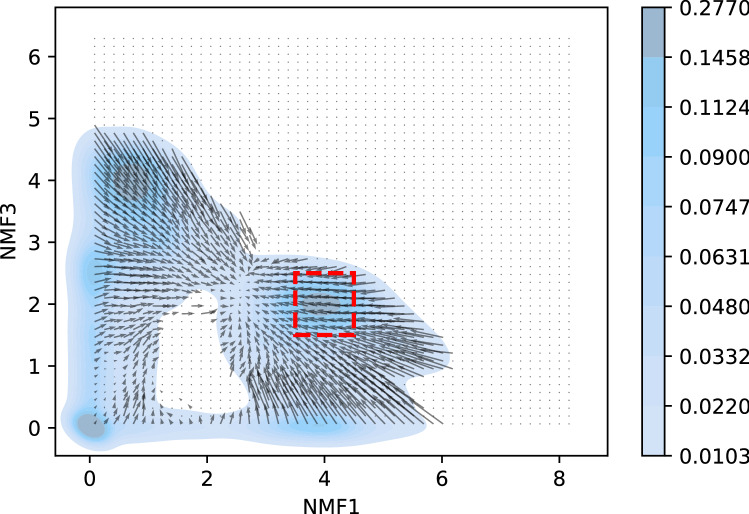
Average velocity in reduced space. The length of the arrow is proportional to the average velocity in the reduced space. The background color indicates the density of the samples. The velocity at cells with less than 100 samples was omitted to reduce the effect of noise. Red dashed rectangle represents the area used to investigate velocity distribution in Fig. 10.

To investigate these dynamics more carefully, we plotted the dynamics in the NMF1–NMF3 space in Fig. 9. In this figure, the space is divided into $50 \times 50$ cells and the average velocity is calculated in each cell. First, the dynamics is very slow at (NMF1, NMF3)$\sim (0,0)$. The protein in this region was unfolded, and there was a large degree of freedom in motion. However, almost all motions did not contribute to folding and were ignored in the projection to the NMF1–NMF3 plane. Therefore, the velocity in the projected state becomes very slow. This result is consistent with that of our previous study. Second, there were two fixed points, (NMF1, NMF3)$\sim (2.0,0.0)$ and (2.5, 2.5). The former fixed point appeared unstable, whereas the latter was stable. However, when we investigated the 3D plot shown in Fig. 8, the saddle point was not a fixed point in 3D space; When NMF4 was large, NMF1 increased at (NMF1, NMF3)$\sim (2.0,0.0)$, and when NMF4 was small, it decreased. By taking the average over NMF4, we obtained this “saddle”. However, the latter fixed point was close to the stable fixed point in 3D space. Third, Fig. 9 shows two paths to reach the folded state. When NMF1 was larger than 2, the mean velocity headed to the fixed point, and the system approached straight to the stable state. As NMF1 and NMF3 scores increase when V9–K32 and F10–L28 create cycles, respectively, this result suggested that in the first path, V9 and K32 made connections to form a cycle, after which F10 and L28 became close. However, when both NMF1 and NMF3 were small, the system detoured around the area (NMF1, NMF3) $\sim (2.0,1.0)$. NMF3 began to increase when the NMF1 score was kept lower than 1 in the first stage, and the NMF1 score began to increase after NMF3 $\gtrsim 1.0$. In the second stage of this path, the score of NMF3 was almost constant. In this path, the connection between V9 and K32 emerged after the formation of a loop that included F10–L28.

**Figure 10:**
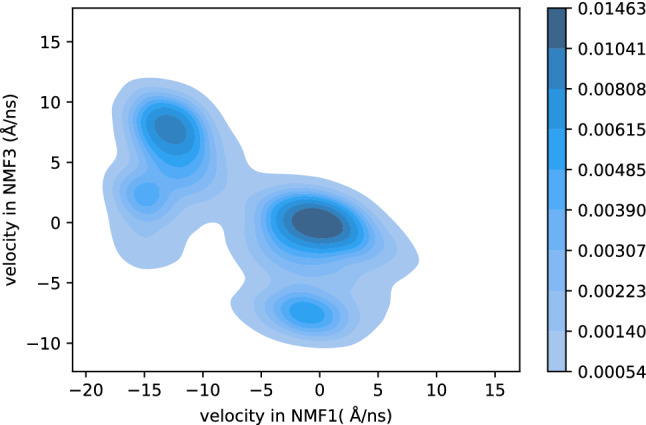
Distribution of velocity in the dense region (3.5 < NMF1 < 4.5, 1.5 < NMF3 < 2.5), depicted by a red dashed rectangle in Fig. 9.

Before concluding this subsection, we note that the information obtained by the mean velocity is limited because there is a large anisotropy in the motion in reduced space. To understand this anisotropy, we investigate the distribution of velocities in the NMF1 and NMF3 direction in the region 3.5 < NMF1 < 4.5 and 1.5< NMF3 <2.5, which corresponds to the high-density region in Fig. 9, depicted by a red dashed rectangle. The result is shown in Fig. 10. From this figure, we found that the distribution of the velocity has several peaks. First, there is a large peak at $(v_{NMF1}, v_{NMF3}) \sim (0,0)$, where $v_{NMF1}$ and $v_{NMF3}$ represent the velocity in the NMF1 and the NMF3 direction, respectively. The motion of these points is slow, which results in a large density in this region. The second largest peak appears at $(v_{NMF1}, v_{NMF3}) \sim (-13,8)$, which makes the average $v_{NMF1}$ to be negative. This multi-peak structure becomes invisible when we describe the dynamics using the mean velocity. However, the discussion based on mean velocity sheds light on the complicated dynamics of protein folding. We also note that the dynamics of protein is stochastic in finite temperature, and there may be trajectories which show another path of folding. For example, there are small number of samples at (NMF1, NMF3)$\sim (2.0,1.0)$ in Fig. 9, which suggests that the straight motion from (NMF1, NMF3)$\sim (0,0)$ to (4.0, 2.0) would be possible. However, low density in this region suggests that such movement rarely occurs.

## Discussion

In this study, we performed TDA of MD trajectories on the folding of the villin headpiece. Using PH and dimensional reduction by NMF, we found that the topological features of this protein could be characterized by several pairs of residues. We also investigated the dynamics of this protein and found two paths for the folding process.

We carried out analyses using the point of residues, without chemical information such as hydropathy, ionization, or hydrogen bonding. Using this information, we will obtain a deeper understanding of the folding process. For example, F6, F10, and F17 form a hydrophobic core, which plays an important role in determining the tertiary structure^26^. Among these three residues, F10 and F17 appeared to be important for determining the tertiary structure, but F6 did not. Vermeulen et al. claimed that the sequence from P21 to W23 plays an essential role in determining the structure of proteins^27^. The importance of the edge L20–Q25 in NMF4 appears to be consistent with this claim, but in our results, P21, L22, and W23 are not important residues. These results indicate that careful investigation is needed when combining the characteristic structure obtained through our method with chemical interactions between residues.

Our method has scope for improvement. In this method, the key idea is to treat volume optimal cycles as “text.” In this approach, we describe a set of volume optimal cycles as the “text” that describes the topology embedded in the dataset. From this viewpoint, other text-mining techniques are applicable to describe the “shape” of proteins. For example, in this study the information of volume optimal cycles is vectorized by a “bag of simplices,” similar to a “bag of words” in text mining. However, in this approach, we only use a small amount of information that is provided by PH. For example, we failed to capture the formation process of helices, and the relation between our study and preceding studies, in which the order of helix formation plays a critical role^13,14^, is unclear. However, as Xia et al. has shown^7^, PH can capture the formation of $\alpha$-helix. If we combine other methods with PH, the relation between our finding and helix formation can be determined. For example, distributed embedding methods, such as Word2Vec or fasttext, have shown excellent performance in natural language processing^28,29^. These methods may be applicable to vectorize PH, although slight modifications are needed. Another important challenge is time series analysis combined with PH. Applications of PH for time series analysis have been reported in several studies^30,31^, but are currently under development. Our vectorization method enables us to apply well-established time-series analysis techniques, such as the state space model, which provides new insights into protein dynamics.

## References

[1] Cohen FE, Kelly JW (2003). Therapeutic approaches to protein-misfolding diseases. Nature.

[2] Maisuradze GG, Liwo A, Scheraga HA (2009). Principal component analysis for protein folding dynamics. J. Mol. Biol..

[3] Jain A, Stock G (2014). Hierarchical folding free energy landscape of HP35 revealed by most probable path clustering. J. Phys. Chem. B.

[4] Das P, Moll M, Stamati H, Kavraki LE, Clementi C (2006). Low-dimensional, free-energy landscapes of protein-folding reactions by nonlinear dimensionality reduction. Proc. Natl. Acad. Sci..

[5] Munch E (2017). A users guide to topological data analysis. J. Learn. Anal..

[6] Yao Y (2009). Topological methods for exploring low-density states in biomolecular folding pathways. J. Chem. Phys..

[7] Xia K, Wei G-W (2014). Persistent homology analysis of protein structure, flexibility and folding. Int. J. Numer. Methods Biomed. Eng..

[8] Xia K, Wei G-W (2015). Multidimensional persistence in biomolecular data. J. Comput. Chem..

[9] Cang Z, Wei G-W (2017). TopologyNet: Topology based deep convolutional and multi-task neural networks for biomolecular property predictions. PLoS Comput. Biol..

[10] Ichinomiya T, Obayashi I, Hiraoka Y (2020). Protein-folding analysis using features obtained by persistent homology. Biophys. J ..

[11] Beauchamp KA, McGibbon R, Lin Y-S, Pande VS (2012). Simple few-state models reveal hidden complexity in protein folding. Proc. Natl. Acad. Sci..

[12] Piana S, Lindorff-Larsen K, Shaw DE (2012). Protein folding kinetics and thermodynamics from atomistic simulation. Proc. Natl. Acad. Sci. USA.

[13] Harada R, Kitao A (2012). The fast-folding mechanism of Villin headpiece subdomain studied by multiscale distributed computing. J. Chem. Theory Comput..

[14] Wang E, Tao P, Wang J, Xiao Y (2019). A novel folding pathway of the Villin headpiece subdomain HP35. Phys. Chem. Chem. Phys..

[15] Michael S, Pande V (2000). Screen savers of the world unite!. Science.

[16] Edelsbrunner H, Letscher D, Zomorodian A (2002). Topological persistence and simplification. Discrete Comput. Geom..

[17] Obayashi I (2018). Volume-optimal cycle: Tightest representative cycle of a generator in persistent homology. SIAM J. Appl. Algebra Geom..

[18] Cohen-Steiner, D., Edelsbrunner, H. & Harer, J. Stability of persistence diagrams. In *Proceedings of the Twenty-First Annual Symposium on Computational Geometry, SCG ’05* 263–271. 10.1145/1064092.1064133 (Association for Computing Machinery, 2005).

[19] Homcloud. https://homcloud.dev/.

[20] Lee DD, Seung HS (1999). Learning the parts of objects by non-negative matrix factorization. Nature.

[21] Hutchins LN, Murphy SM, Singh P, Graber JH (2008). Position-dependent motif characterization using non-negative matrix factorization. Bioinformatics.

[22] Brunet J-P, Tamayo P, Golub TR, Mesirov JP (2004). Metagenes and molecular pattern discovery using matrix factorization. Proc. Natl. Acad. Sci..

[23] R Core Team (2021). R: A Language and Environment for Statistical Computing.

[24] Gaujoux R, Seoighe C (2010). A flexible R package for nonnegative matrix factorization. BMC Bioinform..

[25] Pedregosa F (2011). Scikit-learn: Machine Learning in Python. J. Mach. Learn. Res..

[26] Frank B, Vardar D, Buckley D, James McKnight C (2002). The role of aromatic residues in the hydrophobic core of the Villin headpiece subdomain. Protein Sci..

[27] Vermeulen W (2006). Identification of the PXW sequence as a structural gatekeeper of the headpiece C-terminal subdomain fold. J. Mol. Biol..

[28] Mikolov, T., Chen, K., Corrado, G. & Dean, J. Efficient estimation of word representations in vector space. In *1st International Conference on Learning Representations, ICLR 2013—Workshop Track Proceedings* (2013).

[29] Bojanowski P, Grave E, Joulin A, Mikolov T (2017). Enriching word vectors with subword information. Trans. Assoc. Comput. Linguist..

[30] Perea JA, Harer J (2015). Sliding windows and persistence: An application of topological methods to signal analysis. Found. Comput. Math..

[31] Pereira CMM, De Mello RF (2015). Persistent homology for time series and spatial data clustering. Expert Syst. Appl..

